# Supplementary material for: Safety and Immunogenicity of OVX836, a Nucleoprotein-Based Universal Influenza Vaccine, Co-Administered with Fluarix® Tetra, a Seasonal Hemagglutinin-Based Vaccine
Source: Vaccines (Basel). 2025 May 23;13(6):558. doi: 10.3390/vaccines13060558 (PMC12197426; doi:10.3390/vaccines13060558)
Supplement: Supplementary file 1 [file vaccines-13-00558-s001.zip › vaccines-3627798-supplementary/Vaccines-3627798_Supplementary S1.pdf]

Supplementary S1: Eligibility criteria

Inclusion criteria:

The following criteria were checked at the time of screening (Day 1). The subject could only be included in the study if all inclusion criteria were fulfilled:

1. Written informed consent.
2. Healthy male or female subjects, as determined by medical history and medical examination.
3. Between the age of 18 and 55 years, inclusive.
4. Subjects who had fully been vaccinated with licensed SARS-CoV-2 (COVID-19) vaccine(s) according to national recommendations for the corresponding population group, in vigour at the moment the study started.
5. Reliable and willing to make themselves available for the duration of the study, and willing and able to follow study procedures.
6. Ability and technical possibility for completing an electronic diary (e-diary) and electronic patient-reported outcome (ePRO).

Exclusion criteria:

The following criteria were checked at the time of screening (Day 1). If any exclusion criterion applied, the subject could not be included in the study. Subjects with temporary exclusion criteria could be enrolled at a later point in time when the exclusion criterion had resolved:

1. Subjects with a body mass index (BMI) $\leq$19 kg/m² or $\geq$35 kg/m² on the day of vaccination.
2. Previous influenza vaccination within 6 months before the day of vaccination or planned to receive during the study duration.
3. Any known or suspected immunodeficient conditions.
4. Past or current history of significant autoimmune diseases, as judged by the Investigator.
5. Current history of uncontrolled medical illness such as diabetes, hypertension, heart, renal or hepatic diseases.
6. Known or suspected infection with human immunodeficiency virus (HIV), hepatitis C virus (HCV), or hepatitis B virus (HBV), based upon medical history or physical examination findings.
7. Female subjects: pregnant, breast-feeding or of childbearing potential without appropriate contraceptive methods in place for 2 months before enrolment, or with positive pregnancy test on the day of vaccination. Appropriate contraceptive methods were to be maintained until the end of the trial. Appropriate contraceptive methods were defined by the Clinical Trial Facilitation Group [CTFG] as follow: *"Contraceptive methods that can achieve a failure rate of less than 1% per year when used consistently and correctly are considered as highly effective birth control methods. Such methods include: combined (oestrogen- and progestogen-containing) hormonal contraception associated with inhibition of ovulation (oral, intravaginal, transdermal), progestogen-only hormonal contraception associated with inhibition of ovulation (oral, injectable, implantable intrauterine device, intrauterine*

hormone-releasing system), bilateral tubal occlusion, vasectomized partner and/or sexual abstinence (refraining from heterosexual intercourse).”

8. Having received another vaccination within 3 months prior to the day of study vaccination with live attenuated vaccines, or within 1 month prior to the day of study vaccination with inactivated vaccines, except COVID-19 vaccine.
9. Planning to receive other vaccines during the first 28 days following the study vaccine administration, except COVID-19 vaccine.
10. Having received a COVID-19 vaccination within 2 weeks prior to the day of study vaccination.
11. Planning to receive COVID-19 vaccine during the first week (within 7 days) following the study vaccine administration. An interval of preferably 14 days was recommended. If for scheduling reasons, COVID-19 vaccine had to be given on Day 8, the vaccination had to be administered after completion of the study procedures.
12. Administration of any investigational or non-registered drug or vaccine within 3 months prior to the administration of study vaccines, or planned administration of any such product during the whole study period.
13. History of receiving blood, blood components or immunoglobulins within 3 months prior to the day of vaccination, or planned to receive such product during the whole study period.
14. Presence of an acute febrile illness on the day of vaccination (oral temperature >38.0°C, temporary exclusion criterion).
15. Past or current history of any progressive or severe neurological disorder, seizure disorder or Guillain-Barré syndrome.
16. Behavioural or cognitive impairment, or psychiatric disease that, in the opinion of the Investigator, could interfere with the subject's ability to participate in the study.
17. Past (stopped less than 6 months before enrolment) or current history of alcohol or drug abuse, or current smoking habit above 10 cigarettes per day, or current vaping.
18. Treatment that could affect immune response such as systemic or high dose inhaled corticosteroids (>800μg/day beclomethasone or equivalent; occasional inhaled corticosteroids for asthma therapy are allowed), radiation treatment, cytotoxic drugs, or current or recent (within 30 days before study entry) chronic or prolonged (>10 days) use of systemic non-steroidal anti-inflammatory drugs, interferon, immunomodulators, allergy shots, as judged by the Investigator.
19. History of severe allergic reactions and/or anaphylaxis, or serious adverse reactions to vaccines or allergy to any component of either of the study vaccines, including to egg protein or to kanamycin.
20. Any contraindication to IM administration, as judged by the Investigator, including bleeding disorders such as haemophilia or anticoagulant therapy.
21. Individuals with history of any illness that, in the opinion of the Investigator, could interfere with the results of the study or pose additional risk to the subjects due to participation in the study.
22. Subjects with tattoos in the deltoid region bilaterally, which could interfere with observation of local signs and symptoms or other adverse events at the injection sites. In case of tattoos on one side, injections of study vaccines had to be performed in the contralateral arm.

23. Sponsor employees or Investigator site personnel directly affiliated with this study, and their immediate families. Immediate family was defined as a spouse, parent, child or sibling, whether biological or legally adopted, including children of newly composed families.

The occurrence of one or several of these exclusion criteria after the subject had been enrolled and vaccinated could constitute an elimination criterion for the immunological analyses in the per protocol (PP) cohort. It would however not prevent following-up the concerned subject until the end of the study.
