# Supplementary material for: Safety and Immunogenicity of OVX836, a Nucleoprotein-Based Universal Influenza Vaccine, Co-Administered with Fluarix® Tetra, a Seasonal Hemagglutinin-Based Vaccine
Source: Vaccines (Basel). 2025 May 23;13(6):558. doi: 10.3390/vaccines13060558 (PMC12197426; doi:10.3390/vaccines13060558)
Supplement: Supplementary file 1 [file vaccines-13-00558-s001.zip › vaccines-3627798-supplementary/Vaccines-3627798_Supplementary S2.pdf]

Supplementary S2: Immunoassays

ELISPOT assay for peripheral blood mononuclear cells (PBMCs)

The ELISPOT method was used to measure the numbers of PBMCs that secreted interferon gamma (IFNγ) upon nucleoprotein (NP) in vitro stimulation (NP-specific IFNγ SFCs).

After PBMC thawing, resuspension in culture medium, and overnight resting, $2x10^5$ PBMCs/well in triplicate wells were incubated for 24 hours with the NP peptide pool (>80% purity), at a final concentration of 4µg per peptide/mL. The NP peptide pool was composed of 122 x 15-mer peptides overlapping by 11 amino acids and spanning the amino-acid sequence of the NP from influenza A/WSN/33 (H1N1) strain. As negative control, cells were incubated in the same conditions without the peptide pool (culture medium + 0.3% dimethyl-sulfoxide [DMSO]). The average SFCs for negative control was subtracted from the average SFC for the test wells and result was reported as SFC per million PBMCs for each clinical sample.

The ELISPOT assay was qualified using quality control (QC) samples. The method was specific. The Limit of Detection (LOD) was 29 SFC/million PBMC and the estimated lower limit of quantification (LLOQ) was 82 SFC/million PBMC. The estimated upper limit of quantification (ULOQ) was 3743 SFC/million PBMC (based on anti-CD3 positive control as no sample exhibiting high NP signal was available for the assay qualification). The method presented acceptable within- and between- run precision for samples >LLOQ (coefficient of variation [CV] within-run was <30%, CV between-run <50% on at least 3 days, with 3 replicates of triplicate wells per day).

One internal QC was tested on each plate in parallel to the evaluation of clinical samples, to validate the plate according to predefined criteria (if criteria not fulfilled, the plate was not valid and a retest was performed). Data below LOD on clinical samples were reported as 15 SFC/million PBMC (1/2 LOD) for the statistical analyses.

Flow cytometry – Intracellular staining (ICS) for PBMC

After thawing of PBMC samples, the cells were washed with culture medium, then incubated *in vitro* with the relevant antigen versus corresponding medium in the presence of costimulatory antibodies to CD28 and CD49d for two hours. Then Brefeldin A, a cytokine secretion blocker, was added for a subsequent overnight incubation. This step ensured the inhibition of cytokine secretion and its accumulation in the expressing cells. The day after, the cells were stained using fluorochrome-conjugated antibodies to phenotypic (CD3, CD4 and CD8) markers and activation/cytokine (IFNγ, IL-2 and TNFα) markers. The cell samples were then analysed in flow cytometry.

The relevant antigen was the same peptide pool used in ELISPOT Assay and made of 122 x 15-mer peptides overlapping by 11 amino-acid and spanning the sequence of the NP from influenza A/WSN/33 (H1N1) strain. The concentration of stimulation was 4µg per peptide/mL and negative control including 0.3% DMSO was tested for each sample.

The NP-specific CD4+ and CD8+ T-cells were determined by cytometry as the percentage of CD3+CD4+ and CD3+CD8+ events expressing one cytokine or a combination of cytokines (IFNγ, IL-2 and TNFα) in respectively the total CD3+CD4+ and CD3+CD8+ events after in vitro stimulation with relevant antigen subtracted by the corresponding signal of the same sample obtained after in vitro stimulation with medium + DMSO (blank). The ICS results were reported as the frequencies (%) of NP-specific CD4+/CD8+ T-cells per respectively CD4+ T-cells and CD8+ T-cells. Final results below 0.0001% were set at 0.0001%.

Assay for Anti-NP serum IgG Antibody

The method used to measure anti-NP serum IgG antibody was an indirect ELISA including the following steps: 1) coating of NP (Osivax R&D batch) on the wells of the plate, 2) blocking of non-specific sites using bovine serum albumin, 3) incubation with the clinical samples after two-fold serial dilutions (first dilution 1:800), 4) addition of the secondary anti-human IgG antibody conjugated to Horse Radish Peroxidase, and 5) revelation of the signal using 3,3',5,5'-tetramethylbenzidine (TMB) substrate. The anti-NP IgG titre for a serum sample was defined as the highest tested dilution that gives an optical density >2 fold the signal of the negative control tested on each plate.

The method was qualified using different positive (QC+) and negative (QC-) serum controls. The method was specific, selective and demonstrated acceptable within-run precision (n=6, CV <20% on optical densities; QC+ titre: 6400 ± 1 dilution) and between-run precision (CV <30% on optical densities; QC+ titre: 6400 ± 1 dilution, n=3 tests on 3 different days with 2 operators). The stability of the QC samples upon 3 freeze-thaw cycles and their stability after 5 months at -20°C was also checked. The QC were used in parallel to clinical samples, to validate each plate according to predefined criteria (if criteria not fulfilled, the plate was not valid and a retest was performed).

Serum hemagglutination-inhibition assay (HAI)

The HAI assay is the most used reference method for the assessment of anti-HA antibody levels. The method is based on the ability of influenza virus to agglutinate red blood cells (RBCs) and the ability of anti-HA antibodies to inhibit this agglutination. The biological relevance of the assay is rooted in the fact that the agglutination is mediated by the receptor binding site of the HA1 domain which is also a common target for neutralizing antibodies.

In brief, serum samples are treated with receptor destroying enzyme to remove nonspecific agglutination that may occur. Sera sample dilutions are then added with standardized amount of HA (4 hemagglutination assay units) and the hemagglutination inhibition titre is expressed as the reciprocal of the highest serum dilution able to prevent the hemagglutination of the RBCs. HAI seroconversion is assessed by comparing the inhibition titre post-vaccination to pre-vaccination titre, and was actually defined as a negative pre-vaccination HAI titre and post-vaccination HAI titre ≥1:40, or a fourfold increase in HAI titre between pre- and post-vaccination timepoints (according to the Note for Guidance on Harmonization of Requirements for Influenza Vaccines, CPMP/BWP/214/96, 12 March 1997). Relevant and pre-established positive and negative QC serum samples were included in the testing runs and used to certify the results.

The assay was validated before initiating clinical sample testing. The parameters examined were: linearity, relative accuracy, precision/repeatability (intra-assay precision), intermediate precision, limit of quantitation and range, and specificity. The validation experiments were performed for the following viruses, corresponding to the strains included in the seasonal influenza vaccine used in the study:

- A/Victoria/2570/2019 (H1N1)
- A/Darwin/9/2021 (H3N2)
- B/Austria/1359417/2021 (B/Vic)
- B/Phuket/3073/2013 (B/Yam)

Using selected sheep or ferret sera as positive sera against the relevant strains and human serum depleted of IgA/IgM/IgG as negative control, the HAI assay validation resulted to be linear for (i)

A/Victoria/2570/2019 (H1N1) strain, up to the 1:512 dilution, for (ii) A/Darwin/9/2021 (H3N2) strain, up to the 1:1024 dilution, (iii) B/Austria/1359417/2021 (B/Vic) strain, up to the 1:256 dilution, and (iv) B/Phuket/3073/2013 (B/Yam) strain, up to the 1:128 dilution. The relative accuracy (defined as the agreement between the expected and observed titres across the dilution series) was within 50% - 200% of the expected titre, repeatability (% of geometric coefficient of variation [GCV]<65.5%) and intermediate precision (% of GCV<129%) were demonstrated for the four strains. The limits of quantification and assay ranges were established. The performance of the HAI assay is detailed in the corresponding validation report.
