# Supplementary material for: Safety and Immunogenicity of OVX836, a Nucleoprotein-Based Universal Influenza Vaccine, Co-Administered with Fluarix® Tetra, a Seasonal Hemagglutinin-Based Vaccine
Source: Vaccines (Basel). 2025 May 23;13(6):558. doi: 10.3390/vaccines13060558 (PMC12197426; doi:10.3390/vaccines13060558)
Supplement: Supplementary file 1 [file vaccines-13-00558-s001.zip › vaccines-3627798-supplementary/Vaccines-3627798_Supplementary S3.pdf]

Supplementary S3: Statistical methods

General principles

SAS (Version 9.4; SAS Institute, Cary, North Carolina, USA) was used for the statistical analyses.

The main analyses were descriptive. For inferential analyses, a significance level of 5% (α=5%) was considered. The trial was powered for its immunogenicity primary endpoint only. No adjustment for multiplicity of endpoints was performed. Therefore, all the other inferential tests leading to p-values lower than 5% (even after application of Bonferroni's correction for multiplicity in each individual test) were to be considered with caution, as indicative only of potential differences.

Missing data were not replaced nor extrapolated, with the following exception, immunological parameters below the lower limit of quantification or the limit of detection were imputed by half their respective value.

Analysis of demographics and baseline characteristics

Demographics and baseline characteristics of the subjects were analysed descriptively. The balance between the three treatment groups at baseline was analysed as follows:

- Continuous variables: In case non-normality could not be concluded, a one-way ANOVA with the factor 'treatment group' as classification criterion was used to evaluate the balance between treatment groups. For the ANOVA intergroup comparisons, an adjusted significance α threshold equal to 0.017 was used. If the Kolmogorov-Smirnov test yielded a significant p-value implying non-normality, the intergroup comparisons were performed using a Kruskal-Wallis test to check if there was a difference between the treatment groups. If the Kruskal-Wallis test was statistically significant, then Bonferroni-adjusted p values (α=0.017) by post-hoc Mann-Whitney's tests were used for the three intergroup comparisons.
- Categorical variables: The balance between the treatment groups was evaluated by using either Fisher's exact tests or chi-square tests. The choice of test chi-square/Fisher's exact test was made while programming based on expected frequency $\geq$5/<5.

Analysis of immunogenicity

For each of the immunogenicity endpoints a descriptive analysis by treatment group and visit was performed for absolute values as well as ratio and differences from baseline. Taking into account the central limit theorem and the fact that we had 50-60 subjects evaluable per group (>30 in all cases), parametric tests were used for all inferential tests. If normality was not achieved, additional non-parametric tests were used as well. The following definitions applied for geometric mean titre (GMT), fold rise and geometric mean ratio (GMR):

- GMT = EXP[Σlog10(Ti)/N] where i = 1, 2, …, N, where N denotes the number of subjects within each treatment group and Ti is the per subject antibody titre.
- Fold Rise (Flr) = [TD8 or TD29]/[TD1], where TD8 and TD29 are the per subject antibody titres on 8 Days (PBMC subset) and 29 Days respectively. TD1 = per subject antibody titres on Day 1 (pre-vaccination).
- GMR = EXP[Σlog(FIri)/N] where i = 1, 2, …, N, where N denotes the number of subjects within each treatment group

The anti-NP IgG titres and the HAI titres for the four different strains were summarized using descriptive statistics including GMTs and GMRs with 95% CI for all timepoints. Furthermore, the number and percentage of subjects with a four-fold increase in anti-NP IgG titre on Day 8, Day 29 with respect to pre-injection baseline (Day 1) were calculated. The intergroup

comparisons for the number of subjects with four-fold increase in anti-NP IgG at Day 8 and at Day 29 were performed using chi-square or Fisher's exact tests, as appropriate. Continuous variables were analysed using ANOVA for repeated measures, with the factor 'time' or 'treatment group' and interaction of both factors as fixed effects and subject as random effect. Intergroup comparisons at each post-vaccination timepoint were performed using ANOVA for continuous variables, followed when significant by post-hoc tests adjusted for multiplicity (Bonferroni) for continuous variables.

The absolute values of NP-specific IFNγ T-cells (spot forming cell [SFC]/million peripheral blood mononuclear cell [PBMC]) at Day 1 and Day 8, the change (mean difference and mean ratio) at Day 8 from baseline (Day 1) of NP-specific IFNγ T-cells frequencies were summarized using descriptive statistics. Intergroup comparison for absolute values of NP-specific IFNγ T-cells (SFC/million PBMC) between Day 1 and Day 8 were performed using ANOVA for continuous variables, followed when significant by post-hoc tests adjusted for multiplicity (Bonferroni). The change from baseline, in terms of difference and mean ratio, of NP-specific IFNγ T-cells frequencies between the 3 treatment groups was analysed at Day 8 using ANOVA, followed when significant by post-hoc tests adjusted for multiplicity (Bonferroni). In case non-normality was concluded, a non-parametric Friedman test was used instead of ANOVA.

The absolute values and changes from baseline (in terms of difference) of NP-specific CD4+ and C8+ T-cells expressing the different combinations of 3 markers (IFNγ, IL-2 and/or TNFα) were summarized using descriptive statistics. Intergroup comparison for absolute values of NP-specific CD4+ and C8+ T-cells frequencies positive for each of the possible combinations were performed using ANOVA for continuous variables, followed when significant by post-hoc tests adjusted for multiplicity (Bonferroni). The change from baseline, in terms of difference, of NP-specific CD4+ and C8+ T-cells frequencies positive for each of the combinations was analysed at Day 8 using ANOVA including baseline frequency as covariate and treatment as factor, followed when significant by post-hoc tests adjusted for multiplicity (Bonferroni). In case non-normality was concluded, a non-parametric Friedman test was used instead of ANOVA.

<u>Analysis of safety</u>

The number and percentage of subjects reporting each type and at least one local/systemic sign or symptom during 7 days after vaccine administration were summarized for each of the following types: any, mild (grade 1), moderate (grade 2) and severe (grade ≥3) local/systemic signs or symptoms. Causal relationship of systemic symptoms with the study vaccination was analysed in two categories: related (certainly related, likely/probably related or possibly related) and not related (not related or unlikely related).

The number and percentage of unsolicited AEs/SAEs (overall, severe, considered related to the vaccines/placebo by the Investigator) were summarized by system organ class (SOC) and preferred term (PT) using the Medical Dictionary for Regulator Activities (MedDRA).

Intergroup comparisons were performed using chi-square or Fisher's exact tests, as appropriate.
