# Supplementary material for: Safety and Immunogenicity of OVX836, a Nucleoprotein-Based Universal Influenza Vaccine, Co-Administered with Fluarix® Tetra, a Seasonal Hemagglutinin-Based Vaccine
Source: Vaccines (Basel). 2025 May 23;13(6):558. doi: 10.3390/vaccines13060558 (PMC12197426; doi:10.3390/vaccines13060558)
Supplement: Supplementary file 1 [file vaccines-13-00558-s001.zip › vaccines-3627798-supplementary/Vaccines-3627798_Supplementary S4.pdf]

Supplementary S4: Raw immunogenicity data

Hemagglutination inhibition assay

HAI antibody titres at pre-injection baseline (Day 1) and Day 29, for the four strains included in the IIV, in the three treatment groups (Per Protocol cohort)

Time	Treatment	N	GMT	Median	Min	Max	LL 95% CI	UL 95% CI
A/Victoria/2570/2019 (H1N1) pdm09								
Day 1	IIV + OVX836 480µg	49	26.35	28.28	5.00	640.00	18.68	37.18
	IIV + placebo	48	15.99	10.00	5.00	226.27	11.88	21.51
	OVX836 480µg + placebo	46	15.60	10.00	5.00	320.00	11.38	21.37
Day 29	IIV + OVX836 480µg	49	179.17	160.00	20.00	1280.00	133.81	239.91
	IIV + placebo	48	112.32	113.14	20.00	1280.00	84.15	149.93
	OVX836 480µg + placebo	46	21.08	12.07	5.00	320.00	15.68	28.34
A/Darwin/9/2021 (H3N2)								
Day 1	IIV + OVX836 480µg	49	41.59	40.00	5.00	452.55	30.72	56.30
	IIV + placebo	48	31.29	28.28	5.00	226.27	24.41	40.11
	OVX836 480µg + placebo	46	21.24	20.00	5.00	80.00	17.07	26.43
Day 29	IIV + OVX836 480µg	49	132.19	160.00	10.00	1280.00	99.89	174.92
	IIV + placebo	48	87.87	80.00	10.00	905.10	65.34	118.18
	OVX836 480µg + placebo	46	19.78	20.00	5.00	320.00	14.86	26.31
B/Austria/1359417/2021								
Day 1	IIV + OVX836 480µg	49	7.22	5.00	5.00	40.00	6.18	8.45
	IIV + placebo	48	6.44	5.00	5.00	28.28	5.67	7.31
	OVX836 480µg + placebo	46	7.34	5.00	5.00	80.00	6.04	8.93
Day 29	IIV + OVX836 480µg	49	41.44	40.00	7.07	160.00	32.80	52.35
	IIV + placebo	48	37.21	40.00	5.00	320.00	27.39	50.56
	OVX836 480µg + placebo	46	9.20	5.00	5.00	226.27	7.02	12.07
B/Phuket/3073/2013-like (B/Yamagata lineage)								
Day 1	IIV + OVX836 480µg	49	8.03	5.00	5.00	80.00	6.48	9.95
	IIV + placebo	48	7.33	5.00	5.00	160.00	5.99	8.98
	OVX836 480µg + placebo	46	9.14	5.00	5.00	40.00	7.29	11.45
Day 29	IIV + OVX836 480µg	49	58.61	56.57	10.00	320.00	47.96	71.61
	IIV + placebo	48	51.50	56.57	10.00	640.00	39.45	67.22
	OVX836 480µg + placebo	46	25.64	24.14	5.00	160.00	19.40	33.91

N = number of subjects with results available and valid in the PP-D29; GMT = geometric mean titer; Min= Minimum; Max = Maximum; LL and UL 95%CI = lower and upper limit of the 95% confidence interval.

NP-specific cell-mediated immune response

Number of NP-specific IFNγ spot forming cells (SFCs) per million PBMCs at Day 1 (vaccination day) and Day 8, and differences (absolute and fold-rise) between Day 8 and Day 1 in the three treatment groups (Per Protocol cohort; PBMC subset)

Treatment	Time point		n	Mean	SD	95% CI	Median	Min	Max
IIV + OVX836 480ug (N=24)	Day 1	Absolute value	24	57.8	49.54	36.91, 78.75	44.0	15.0	175.0
	Day 8	Absolute value	22	226.0	193.23	140.33, 311.67	143.5	40.0	680.0
		Difference from Baseline	22	164.3	189.83	80.11, 248.44	111.5	-19.0	655.0
		Fold rise from Baseline	22	6.5	9.21	2.38, 10.55	3.0	0.8	44.7
IIV + placebo (N=23)	Day 1	Absolute value	23	62.7	60.25	36.64, 88.75	37.0	15.0	212.0
	Day 8	Absolute value	19	60.3	49.68	36.37, 84.26	53.0	15.0	178.0
		Difference from Baseline	19	0.5	34.99	-16.34, 17.39	0.0	-111.0	62.0
		Fold rise from Baseline	19	1.3	1.00	0.81, 1.77	1.0	0.3	5.1
OVX836 480ug + placebo (N=24)	Day 1	Absolute value	24	39.5	32.30	25.86, 53.14	31.0	15.0	143.0
	Day 8	Absolute value	23	213.4	152.52	147.43, 279.35	222.0	15.0	715.0
		Difference from Baseline	23	178.4	152.99	112.23, 244.55	183.0	-37.0	700.0
		Fold rise from Baseline	23	9.1	10.39	4.56, 13.54	5.9	0.3	47.7

N = total number of the subjects in the cohort; n = number of subjects with results available and valid in the PP-D29; SD = Standard Deviation; Min = Minimum; Max = Maximum; 95%CI = lower and upper limit of the 95% confidence interval

NP-specific CD4+ T-cell-mediated immune response

Percentage of NP-specific CD4+ T-cells positive at least for one of the three cytokines (among IFNγ, IL-2 and TNFα), at least for IFNγ, for one, two (different combinations) or the three cytokines, and polypositive (at least two cytokines among IFNγ, IL-2 and TNFα) at baseline (Day 1) and Day 8, and absolute differences between Day 8 and Day 1 for the same marker combinations, in the three treatment groups (Per Protocol cohort; PBMC subset)

Treatment	Timepoint	Parameter	n	Mean %	SD %	95% CI (%)	Median %	Min %	Max %
**At least one cytokine**									
IIV + OVX836 480ug (N=24)	Day 1	Absolute value	23	0.027	0.026	0.016, 0.038	0.024	0.000	0.084
	Day 8	Absolute value	17	0.101	0.080	0.060, 0.142	0.078	0.022	0.333
		Difference from Baseline	16	0.079	0.073	0.040, 0.117	0.070	0.000	0.263
IIV + placebo (N=23)	Day 1	Absolute value	20	0.024	0.023	0.013, 0.035	0.021	0.000	0.099
	Day 8	Absolute value	18	0.024	0.031	0.009, 0.040	0.016	0.000	0.129
		Difference from Baseline	17	0.002	0.018	-0.007, 0.012	-0.000	-0.034	0.033
OVX836 480ug + placebo (N=24)	Day 1	Absolute value	24	0.026	0.039	0.010, 0.042	0.017	0.000	0.186
	Day 8	Absolute value	21	0.126	0.089	0.086, 0.167	0.102	0.028	0.323
		Difference from Baseline	21	0.098	0.080	0.062, 0.135	0.071	0.007	0.305
**At least IFNγ**									
IIV + OVX836 480ug (N=24)	Day 1	Absolute value	23	0.025	0.020	0.017, 0.034	0.022	0.000	0.081
	Day 8	Absolute value	17	0.092	0.077	0.052, 0.132	0.064	0.023	0.319
		Difference from Baseline	16	0.064	0.072	0.025, 0.103	0.042	0.004	0.281
IIV + placebo (N=23)	Day 1	Absolute value	20	0.020	0.016	0.013, 0.027	0.014	0.006	0.072
	Day 8	Absolute value	18	0.022	0.025	0.010, 0.035	0.014	0.000	0.111
		Difference from Baseline	17	0.003	0.012	-0.003, 0.009	0.001	-0.014	0.040
OVX836 480ug + placebo (N=24)	Day 1	Absolute value	24	0.017	0.013	0.011, 0.022	0.015	0.000	0.054
	Day 8	Absolute value	21	0.113	0.078	0.078, 0.148	0.091	0.024	0.276
		Difference from Baseline	21	0.096	0.075	0.062, 0.130	0.073	0.022	0.263
**IL2+TNFα+IFNγ+**									
IIV + OVX836 480ug (N=24)	Day 1	Absolute value	23	0.015	0.013	0.009, 0.021	0.013	0.000	0.053
	Day 8	Absolute value	17	0.016	0.012	0.010, 0.023	0.014	0.003	0.050
		Difference from Baseline	16	-0.002	0.012	-0.008, 0.005	-0.003	-0.027	0.029
IIV + placebo (N=23)	Day 1	Absolute value	20	0.013	0.010	0.008, 0.018	0.011	0.003	0.051
	Day 8	Absolute value	18	0.011	0.012	0.005, 0.017	0.007	0.001	0.048
		Difference from Baseline	17	-0.002	0.005	-0.005, 0.001	-0.002	-0.013	0.008
OVX836 480ug + placebo (N=24)	Day 1	Absolute value	24	0.011	0.009	0.007, 0.015	0.009	0.000	0.034
	Day 8	Absolute value	21	0.021	0.016	0.014, 0.028	0.018	0.003	0.054
		Difference from Baseline	21	0.010	0.013	0.004, 0.016	0.006	-0.010	0.036
**IL2+TNFα+IFNγ-**									
IIV + OVX836 480ug (N=24)	Day 1	Absolute value	23	0.002	0.003	0.001, 0.003	0.001	0.000	0.009
	Day 8	Absolute value	17	0.003	0.003	0.001, 0.005	0.002	0.000	0.011
		Difference from Baseline	16	0.001	0.004	-0.001, 0.003	0.001	-0.007	0.011
IIV + placebo (N=23)	Day 1	Absolute value	20	0.004	0.003	0.003, 0.006	0.004	0.000	0.012
	Day 8	Absolute value	18	0.003	0.003	0.002, 0.005	0.003	0.000	0.010
		Difference from Baseline	17	-0.001	0.005	-0.003, 0.001	-0.003	-0.008	0.010
OVX836 480ug + placebo (N=24)	Day 1	Absolute value	24	0.002	0.003	0.001, 0.004	0.001	0.000	0.009
	Day 8	Absolute value	21	0.003	0.005	0.001, 0.005	0.001	0.000	0.018
		Difference from Baseline	21	0.000	0.006	-0.002, 0.003	0.000	-0.009	0.018
**IL2+TNFα-IFNγ+**									
IIV + OVX836 480ug (N=24)	Day 1	Absolute value	23	0.005	0.003	0.004, 0.007	0.006	0.000	0.011
	Day 8	Absolute value	17	0.029	0.028	0.015, 0.043	0.024	0.002	0.120
		Difference from Baseline	16	0.025	0.027	0.010, 0.039	0.020	-0.008	0.112
IIV	Day 1	Absolute value	20	0.003	0.003	0.002, 0.005	0.003	0.000	0.010
	Day 8	Absolute value	18	0.005	0.005	0.002, 0.007	0.003	0.000	0.018

Treatment	Timepoint	Parameter	n	Mean %	SD %	95% CI (%)	Median %	Min %	Max %
+ placebo (N=23)		Difference from Baseline	17	0.002	0.005	-0.001, 0.004	0.001	-0.004	0.017
OVX836 480ug + placebo (N=24)	Day 1	Absolute value	24	0.004	0.004	0.002, 0.006	0.003	0.000	0.016
	Day 8	Absolute value	21	0.035	0.025	0.024, 0.046	0.032	0.005	0.094
		Difference from Baseline	21	0.031	0.024	0.020, 0.042	0.023	0.004	0.093
**IL2-TNFα+IFNγ+**									
IIV + OVX836 480ug (N=24)	Day 1	Absolute value	23	0.004	0.004	0.002, 0.006	0.002	0.000	0.016
	Day 8	Absolute value	17	0.004	0.003	0.002, 0.005	0.004	0.000	0.010
		Difference from Baseline	16	-0.000	0.004	-0.002, 0.002	0.001	-0.010	0.005
IIV + placebo (N=23)	Day 1	Absolute value	20	0.002	0.003	0.001, 0.004	0.001	0.000	0.015
	Day 8	Absolute value	18	0.002	0.002	0.001, 0.003	0.001	0.000	0.008
		Difference from Baseline	17	-0.000	0.004	-0.002, 0.002	0.001	-0.014	0.006
OVX836 480ug + placebo (N=24)	Day 1	Absolute value	24	0.003	0.003	0.002, 0.004	0.002	0.000	0.009
	Day 8	Absolute value	21	0.007	0.006	0.004, 0.010	0.006	0.000	0.022
		Difference from Baseline	21	0.004	0.006	0.002, 0.007	0.004	-0.009	0.019
**IL2+TNFα-IFNγ-**									
IIV + OVX836 480ug (N=24)	Day 1	Absolute value	23	0.002	0.002	0.001, 0.003	0.000	0.000	0.009
	Day 8	Absolute value	17	0.005	0.006	0.002, 0.008	0.004	0.000	0.017
		Difference from Baseline	16	0.005	0.006	0.001, 0.008	0.003	-0.004	0.017
IIV + placebo (N=23)	Day 1	Absolute value	20	0.002	0.003	0.001, 0.004	0.001	0.000	0.009
	Day 8	Absolute value	18	0.003	0.004	0.001, 0.005	0.002	0.000	0.013
		Difference from Baseline	17	0.001	0.005	-0.002, 0.003	0.000	-0.006	0.013
OVX836 480ug + placebo (N=24)	Day 1	Absolute value	24	0.003	0.005	0.001, 0.005	0.000	0.000	0.018
	Day 8	Absolute value	21	0.011	0.010	0.006, 0.015	0.008	0.000	0.041
		Difference from Baseline	21	0.007	0.012	0.002, 0.012	0.008	-0.011	0.041
**IL2-TNFα+IFNγ-**									
IIV + OVX836 480ug (N=24)	Day 1	Absolute value	23	0.007	0.013	0.001, 0.013	0.000	0.000	0.053
	Day 8	Absolute value	17	0.005	0.005	0.003, 0.008	0.004	0.000	0.014
		Difference from Baseline	16	0.002	0.008	-0.002, 0.006	0.004	-0.015	0.013
IIV + placebo (N=23)	Day 1	Absolute value	20	0.004	0.007	0.001, 0.008	0.000	0.000	0.029
	Day 8	Absolute value	18	0.005	0.007	0.001, 0.009	0.000	0.000	0.026
		Difference from Baseline	17	0.002	0.009	-0.003, 0.007	0.000	-0.018	0.024
OVX836 480ug + placebo (N=24)	Day 1	Absolute value	24	0.011	0.031	-0.002, 0.024	0.002	0.000	0.153
	Day 8	Absolute value	21	0.005	0.006	0.002, 0.007	0.002	0.000	0.022
		Difference from Baseline	21	-0.007	0.033	-0.023, 0.008	0.000	-0.147	0.022
**IL2-TNFα-IFNγ+**									
IIV + OVX836 480ug (N=24)	Day 1	Absolute value	23	0.002	0.003	0.001, 0.003	0.002	0.000	0.010
	Day 8	Absolute value	17	0.043	0.041	0.022, 0.064	0.034	0.003	0.139
		Difference from Baseline	16	0.041	0.042	0.019, 0.063	0.025	0.001	0.137
IIV + placebo (N=23)	Day 1	Absolute value	20	0.002	0.003	0.001, 0.004	0.001	0.000	0.010
	Day 8	Absolute value	18	0.006	0.011	0.000, 0.012	0.003	0.000	0.050
		Difference from Baseline	17	0.004	0.012	-0.002, 0.010	0.001	-0.005	0.049
OVX836 480ug + placebo (N=24)	Day 1	Absolute value	24	0.001	0.002	0.000, 0.002	0.000	0.000	0.008
	Day 8	Absolute value	21	0.050	0.041	0.031, 0.069	0.037	0.008	0.166
		Difference from Baseline	21	0.049	0.041	0.030, 0.068	0.035	0.008	0.166
**Polypositive (at least two cytokines among the three)**									
IIV + OVX836 480ug (N=24)	Day 1	Absolute value	23	0.023	0.017	0.016, 0.031	0.019	0.000	0.072
	Day 8	Absolute value	17	0.051	0.038	0.032, 0.071	0.038	0.013	0.170
		Difference from Baseline	16	0.026	0.033	0.009, 0.044	0.019	-0.018	0.130
IIV + placebo (N=23)	Day 1	Absolute value	20	0.022	0.015	0.014, 0.029	0.015	0.003	0.075
	Day 8	Absolute value	18	0.019	0.015	0.011, 0.026	0.014	0.001	0.056
		Difference from Baseline	17	-0.003	0.011	-0.009, 0.003	-0.004	-0.019	0.027
OVX836 480ug	Day 1	Absolute value	24	0.017	0.013	0.012, 0.023	0.014	0.000	0.044
	Day 8	Absolute value	21	0.065	0.044	0.044, 0.085	0.053	0.010	0.160

Treatment	Timepoint	Parameter	n	Mean %	SD %	95% CI (%)	Median %	Min %	Max %
**+ placebo (N=24)**		**Difference from Baseline**	21	0.047	0.040	0.029, 0.065	0.037	0.003	0.146

N = total number of the subjects in the cohort; n = number of subjects with results available and valid in the PP-D29; SD = standard deviation; Min = Minimum; Max = Maximum; 95%CI = lower and upper limit of the 95% confidence interval.

NP-specific CD8+ T-cell-mediated immune response

Percentage of NP-specific CD8+ T-cells positive at least for one of the three cytokines (among IFNγ, IL-2 and TNFα), at least for IFNγ, for one, two (different combinations) or the three cytokines, and polypositive (at least two cytokines among IFNγ, IL-2 and TNFα) at baseline (Day 1) and Day 8, and absolute differences between Day 8 and Day 1 for the same marker combinations, in the three treatment groups (Per Protocol cohort; PBMC subset)

Treatment	Timepoint	Parameter	n	Mean %	SD %	95% CI (%)	Median %	Min %	Max %
**At least one cytokine**									
IIV + OVX836 480ug (N=24)	Day 1	Absolute value	23	0.047	0.067	0.018, 0.076	0.023	0.000	0.257
	Day 8	Absolute value	16	0.035	0.026	0.021, 0.049	0.033	0.000	0.075
		Difference from Baseline	15	0.002	0.030	-0.015, 0.019	0.007	-0.064	0.043
IIV + placebo (N=23)	Day 1	Absolute value	20	0.052	0.063	0.023, 0.081	0.037	0.000	0.263
	Day 8	Absolute value	18	0.060	0.059	0.031, 0.089	0.050	0.000	0.251
		Difference from Baseline	17	0.004	0.050	-0.021, 0.030	0.004	-0.148	0.097
OVX836 480ug + placebo (N=24)	Day 1	Absolute value	22	0.050	0.072	0.018, 0.082	0.026	0.000	0.302
	Day 8	Absolute value	21	0.064	0.069	0.033, 0.096	0.053	0.000	0.267
		Difference from Baseline	19	0.021	0.063	-0.010, 0.051	0.008	-0.111	0.146
**At least IFNγ**									
IIV + OVX836 480ug (N=24)	Day 1	Absolute value	23	0.044	0.062	0.017, 0.071	0.016	0.000	0.225
	Day 8	Absolute value	16	0.033	0.026	0.019, 0.047	0.034	0.000	0.077
		Difference from Baseline	15	0.000	0.031	-0.017, 0.017	0.003	-0.061	0.048
IIV + placebo (N=23)	Day 1	Absolute value	20	0.054	0.064	0.024, 0.084	0.042	0.000	0.274
	Day 8	Absolute value	18	0.059	0.058	0.030, 0.088	0.052	0.000	0.247
		Difference from Baseline	17	0.001	0.050	-0.024, 0.027	0.002	-0.153	0.092
OVX836 480ug + placebo (N=24)	Day 1	Absolute value	22	0.046	0.065	0.017, 0.075	0.025	0.000	0.277
	Day 8	Absolute value	21	0.061	0.068	0.030, 0.092	0.049	0.000	0.263
		Difference from Baseline	19	0.021	0.057	-0.007, 0.048	0.003	-0.108	0.164
**IL2+TNFα+IFNγ+**									
IIV + OVX836 480ug (N=24)	Day 1	Absolute value	23	0.004	0.006	0.001, 0.007	0.000	0.000	0.020
	Day 8	Absolute value	16	0.005	0.007	0.001, 0.008	0.002	0.000	0.022
		Difference from Baseline	15	0.001	0.005	-0.002, 0.004	0.000	-0.008	0.018
IIV + placebo (N=23)	Day 1	Absolute value	20	0.007	0.009	0.003, 0.011	0.002	0.000	0.028
	Day 8	Absolute value	18	0.011	0.020	0.001, 0.021	0.003	0.000	0.083
		Difference from Baseline	17	0.004	0.017	-0.004, 0.013	0.000	-0.014	0.068
OVX836 480ug + placebo (N=24)	Day 1	Absolute value	22	0.006	0.013	0.000, 0.012	0.002	0.000	0.063
	Day 8	Absolute value	21	0.006	0.014	0.000, 0.013	0.002	0.000	0.062
		Difference from Baseline	19	0.001	0.005	-0.002, 0.003	0.000	-0.005	0.014
**IL2+TNFα+IFNγ-**									
IIV + OVX836 480ug (N=24)	Day 1	Absolute value	23	0.000	0.001	-0.000, 0.001	0.000	0.000	0.005
	Day 8	Absolute value	16	0.001	0.002	-0.001, 0.002	0.000	0.000	0.010
		Difference from Baseline	15	0.000	0.003	-0.001, 0.002	0.000	-0.005	0.010
IIV + placebo (N=23)	Day 1	Absolute value	20	0.000	0.001	-0.000, 0.001	0.000	0.000	0.004
	Day 8	Absolute value	18	0.000	0.000	-0.000, 0.000	0.000	0.000	0.002
		Difference from Baseline	17	-0.000	0.001	-0.001, 0.000	0.000	-0.004	0.002
OVX836 480ug + placebo (N=24)	Day 1	Absolute value	22	0.001	0.001	0.000, 0.001	0.000	0.000	0.006
	Day 8	Absolute value	21	0.001	0.001	0.000, 0.001	0.000	0.000	0.004
		Difference from Baseline	19	0.000	0.002	-0.001, 0.001	0.000	-0.003	0.004
**IL2+TNFα-IFNγ+**									
IIV + OVX836 480ug (N=24)	Day 1	Absolute value	23	0.007	0.014	0.001, 0.013	0.002	0.000	0.056
	Day 8	Absolute value	16	0.005	0.004	0.002, 0.007	0.004	0.000	0.015
		Difference from Baseline	15	0.002	0.005	-0.001, 0.005	0.001	-0.008	0.015
IIV	Day 1	Absolute value	20	0.009	0.012	0.004, 0.015	0.006	0.000	0.043
	Day 8	Absolute value	18	0.010	0.011	0.005, 0.016	0.007	0.000	0.037

Treatment	Timepoint	Parameter	n	Mean %	SD %	95% CI (%)	Median %	Min %	Max %
+ placebo (N=23)		Difference from Baseline	17	-0.002	0.007	-0.005, 0.002	-0.001	-0.018	0.010
OVX836 480ug + placebo (N=24)	Day 1	Absolute value	22	0.009	0.015	0.002, 0.016	0.004	0.000	0.060
	Day 8	Absolute value	21	0.008	0.012	0.003, 0.013	0.004	0.000	0.049
		Difference from Baseline	19	-0.001	0.007	-0.004, 0.003	0.000	-0.020	0.011
**IL2-TNFα+IFNγ+**									
IIV + OVX836 480ug (N=24)	Day 1	Absolute value	23	0.014	0.016	0.007, 0.021	0.012	0.000	0.072
	Day 8	Absolute value	16	0.008	0.008	0.004, 0.013	0.006	0.000	0.030
		Difference from Baseline	15	-0.004	0.010	-0.010, 0.001	-0.004	-0.020	0.019
IIV + placebo (N=23)	Day 1	Absolute value	20	0.011	0.011	0.006, 0.017	0.009	0.000	0.044
	Day 8	Absolute value	18	0.014	0.020	0.004, 0.024	0.005	0.000	0.076
		Difference from Baseline	17	0.005	0.015	-0.003, 0.013	0.000	-0.011	0.053
OVX836 480ug + placebo (N=24)	Day 1	Absolute value	22	0.014	0.018	0.006, 0.022	0.008	0.000	0.068
	Day 8	Absolute value	21	0.011	0.021	0.002, 0.021	0.002	0.000	0.091
		Difference from Baseline	19	-0.001	0.017	-0.009, 0.007	0.000	-0.056	0.023
**IL2+TNFα-IFNγ-**									
IIV + OVX836 480ug (N=24)	Day 1	Absolute value	23	0.004	0.007	0.001, 0.008	0.000	0.000	0.030
	Day 8	Absolute value	16	0.003	0.003	0.001, 0.004	0.002	0.000	0.011
		Difference from Baseline	15	-0.000	0.005	-0.003, 0.003	0.001	-0.010	0.006
IIV + placebo (N=23)	Day 1	Absolute value	20	0.001	0.002	-0.000, 0.002	0.000	0.000	0.009
	Day 8	Absolute value	18	0.004	0.004	0.002, 0.005	0.003	0.000	0.012
		Difference from Baseline	17	0.002	0.003	0.000, 0.004	0.002	-0.006	0.007
OVX836 480ug + placebo (N=24)	Day 1	Absolute value	22	0.006	0.009	0.003, 0.010	0.003	0.000	0.028
	Day 8	Absolute value	21	0.007	0.014	0.000, 0.013	0.001	0.000	0.063
		Difference from Baseline	19	0.001	0.016	-0.007, 0.009	0.000	-0.025	0.061
**IL2-TNFα+IFNγ-**									
IIV + OVX836 480ug (N=24)	Day 1	Absolute value	23	0.002	0.002	0.001, 0.003	0.000	0.000	0.007
	Day 8	Absolute value	16	0.002	0.002	0.000, 0.003	0.001	0.000	0.009
		Difference from Baseline	15	0.000	0.004	-0.002, 0.003	0.000	-0.007	0.009
IIV + placebo (N=23)	Day 1	Absolute value	20	0.002	0.003	0.001, 0.003	0.000	0.000	0.009
	Day 8	Absolute value	18	0.000	0.001	0.000, 0.001	0.000	0.000	0.002
		Difference from Baseline	17	-0.001	0.003	-0.003, 0.000	0.000	-0.008	0.002
OVX836 480ug + placebo (N=24)	Day 1	Absolute value	22	0.001	0.002	0.000, 0.002	0.000	0.000	0.009
	Day 8	Absolute value	21	0.002	0.003	0.000, 0.003	0.000	0.000	0.013
		Difference from Baseline	19	0.001	0.002	-0.000, 0.002	0.000	-0.004	0.004
**IL2-TNFα-IFNγ+**									
IIV + OVX836 480ug (N=24)	Day 1	Absolute value	23	0.022	0.029	0.009, 0.035	0.010	0.000	0.108
	Day 8	Absolute value	16	0.017	0.018	0.007, 0.027	0.013	0.000	0.060
		Difference from Baseline	15	0.001	0.023	-0.012, 0.013	0.001	-0.044	0.032
IIV + placebo (N=23)	Day 1	Absolute value	20	0.029	0.046	0.007, 0.050	0.015	0.000	0.201
	Day 8	Absolute value	18	0.026	0.020	0.017, 0.036	0.026	0.000	0.058
		Difference from Baseline	17	-0.005	0.043	-0.027, 0.017	0.000	-0.159	0.036
OVX836 480ug + placebo (N=24)	Day 1	Absolute value	22	0.019	0.024	0.008, 0.030	0.009	0.000	0.086
	Day 8	Absolute value	21	0.038	0.039	0.020, 0.056	0.024	0.000	0.159
		Difference from Baseline	19	0.021	0.042	0.001, 0.041	0.007	-0.043	0.146
**Polypositive (at least two cytokines among IFNγ, IL-2 and TNFα)**									
IIV + OVX836 480ug (N=24)	Day 1	Absolute value	23	0.024	0.032	0.010, 0.038	0.010	0.000	0.133
	Day 8	Absolute value	16	0.017	0.016	0.009, 0.025	0.012	0.000	0.053
		Difference from Baseline	15	-0.002	0.012	-0.008, 0.005	0.000	-0.024	0.017
IIV + placebo (N=23)	Day 1	Absolute value	20	0.027	0.025	0.016, 0.039	0.022	0.000	0.086
	Day 8	Absolute value	18	0.035	0.045	0.012, 0.057	0.025	0.000	0.196
		Difference from Baseline	17	0.007	0.028	-0.008, 0.021	-0.002	-0.018	0.110
OVX836 480ug	Day 1	Absolute value	22	0.029	0.044	0.009, 0.048	0.017	0.000	0.193
	Day 8	Absolute value	21	0.024	0.044	0.004, 0.045	0.009	0.000	0.203

Treatment	Timepoint	Parameter	n	Mean %	SD %	95% CI (%)	Median %	Min %	Max %
**+ placebo (N=24)**		**Difference from Baseline**	19	-0.001	0.026	-0.013, 0.011	0.002	-0.066	0.035

N = total number of the subjects in the cohort; n = number of subjects with results available and valid in the PP-D29; SD = standard deviation; Min = Minimum; Max = Maximum; 95%CI = lower and upper limit of the 95% confidence interval.

Anti-NP immunoglobulin G humoral response

Anti-NP IgG titers at pre-injection baseline (Day 1), Day 8 (PBMC subset only) and Day 29, and differences (fold-rise) between Day 8 and Day 1, and between Day 29 and Day 1, in the three treatment groups (Per Protocol cohort)

Treatment	Time point		n	Geometric Mean	95% CI of GM	Median	Min	Max
IIV + OVX836 480ug (N=49)	Day 1	Antibody Titer	49	2939.61	2466.62, 3503.28	3200	800	12800
	Day 8	Antibody Titer	24	12800.00	9492.10, 17260.67	12800	3200	51200
		Fold Rise from baseline	24	4.36	2.99, 6.37	4	1	32
	Day 29	Antibody Titer	49	13738.12	11494.75, 16419.32	12800	6400	51200
		Fold Rise from baseline	49	4.67	3.74, 5.84	4	2	32
IIV + placebo (N=48)	Day 1	Antibody Titer	48	3293.77	2723.61, 3983.28	3200	800	12800
	Day 8	Antibody Titer	21	4306.88	3403.16, 5450.58	3200	1600	12800
		Fold Rise from baseline	21	1.18	0.97, 1.44	1	0.5	4
	Day 29	Antibody Titer	48	4271.49	3567.20, 5114.82	3200	1600	25600
		Fold Rise from baseline	48	1.30	1.15, 1.47	1	0.5	4
OVX836 480ug + placebo (N=46)	Day 1	Antibody Titer	46	3058.56	2490.68, 3755.93	3200	800	12800
	Day 8	Antibody Titer	24	14788.51	10969.57, 19936.99	12800	3200	51200
		Fold Rise from baseline	24	4.24	3.00, 5.98	4	2	32
	Day 29	Antibody Titer	46	22019.05	18420.15, 26321.10	25600	6400	102400
		Fold Rise from baseline	46	7.20	5.75, 9.02	8	2	32

N = total number of the subjects in the cohort; n = number of subjects with results available and valid in the PP-D29; GM = geometric mean; Min = Minimum; Max = Maximum; 95%CI = lower and upper limit of the 95% confidence interval.
